# Supplementary material for: Patterns of risk for diabetic retinopathy in the Mumbai slums: The Aditya Jyot Diabetic Retinopathy in Urban Mumbai Slums Study (AJ-DRUMSS) Report 3
Source: PLOS Glob Public Health. 2023 Apr 12;3(4):e0000351. doi: 10.1371/journal.pgph.0000351 (PMC10096465; doi:10.1371/journal.pgph.0000351)
Supplement: S2 Table — (DOCX) [file pgph.0000351.s002.docx]

| Data Dictionary | Description |
| --- | --- |
| Variable |  |
| StatusDM | DM=Diabetes Mellitus; 0=No; 1=Yes;.=missing |
| StatusDR | DR=Diabetic Retinopathy: 0=No; 1=Yes; 2=not available;.=missing |
| Age | In Years;.=missing |
| Sex | F=Female; M=Male |
| Literacy | 0=Illiterate; 1=literate;.=missing |
| Religion | Hindu; Muslim:2; .=missing |
| Occupation | 0=Not Working; 1=Working; 2=Retired; 3=Housewife; .=missing |
| Smoking | 0=No; 1=Yes; .=missing |
| ELC | Diagonal Ear Lobe Crease (ELC);0=Absent; 1=Present; .=missing |
| Polyuria | 0=No; 1=Yes;.=missing |
| Polydypsia | 0=No; 1=Yes; 2=Not Known;.=missing |
| Weightloss | 0=No; 1=Yes;.=missing |
| FHDM | Family History of Diabetes Meillitus; 0=No; 1=Yes; .=missing |
| IHD | Ischemic Heart Disease; 0=No; 1=Yes; .=missing |
| Stroke | 0=No; 1=Yes; .=missing |
| Neuropathy | 0=No; 1=Yes;.=missing |
| Nephropathy | 0=No; 1=Yes; .=missing |
| Abdcircode | Abdominal Circumference Code; 1=Normal (male ?102cm; female ?88cm); 2=Obese (male >102cm; female >88cm);.=missing |
| Treatment | Metformin Treatment; 0= No; 1=Yes;.=missing |
| StatusHTN | Status of Hypertension; 0=No; 1=Yes; .=missing |
| DurHTN | Duration of Hypertension; .=missing, na=not applicable;.=missing |
| BPSystolic | Systolic Blood Pressure;.=missing |
| BPDiastolic | Diastolic Blood Pressure;.=missing |
| DurDM | Duration of Diabetes Mellitus; 0=Newly detected |
| FPGValue | Fasting Glucose Value;.=missing |
| Weight | Weight (Kg);.=missing |
| Height | Height(cm);.=missing |
| BMI | BMI (Kg/m2);.=missing |
| COScore | Central Obesity Score Code; 1=Normal (Femal<0.85/Male<0.95); 2=Central Obesity (Female>=0.85/Male>=0.95); .=missing |
| WHRCO | Waist to Hip Ratio Central Obesity;.=missing |
| Abdcircum | Abdominal Circumference (cm) ; .=missing |
| Hipcirum | Hip Circumference (cm); .=missing |
| AHRatio | Abdominal to Hip Ratio;. = missing |
| DurRX | Duration of Treatment of Diabetes;.=missing |
| VegDiet | Vegetarian Diet; 1=Vegetarian ; 2= Not Vegetarian; .=missing |
| Food | Rice or Wheat Eater or Both; 1=Rice; 2=Wheat;3=Both Rice and Wheat; .=missing |
