# Supplementary material for: Patterns of risk for diabetic retinopathy in the Mumbai slums: The Aditya Jyot Diabetic Retinopathy in Urban Mumbai Slums Study (AJ-DRUMSS) Report 3
Source: PLOS Glob Public Health. 2023 Apr 12;3(4):e0000351. doi: 10.1371/journal.pgph.0000351 (PMC10096465; doi:10.1371/journal.pgph.0000351)
Supplement: S3 Table — (DOCX) [file pgph.0000351.s003.docx]

**Table S3. Variables collected as part of the study**

| **Variables** | **Methodology** | **Mode of Collection/ Equipment used** |
| --- | --- | --- |
| Sex | questionnaire | NA |
| Age | QUESTIONNAIRE |  |
| Hypertension | Questionnaire | PAst Medical REcords |
| Smoking | questionnaire | PAst Medical REcords |
| Ear Lobe Crease | Physical Examination | Presence or Absence noted by Observation |
| Polyuria | questionnaire | PAst Medical REcords |
| POLYDIPSIA | questionnaire | PAst Medical REcords |
| Weight Loss | questionnaire | PAst Medical REcords |
| Ischemic Heart Disease | questionnaire | PAst Medical REcords |
| Stroke | Questionnaire | PAst Medical REcords |
| Neuropathy | Questionnaire | PAst Medical REcords |
| Nephropathy | Questionnaire | PAst Medical REcords |
| Family history of Diabetes | Questionnaire | Recorded Subject’s statement |
| Vegetarian Diet | Questionnaire | Recorded Subject’s statement |
| LiteraCY | Questionnaire | Participant SELF-Reported * |
| Religion | Questionnaire | Recorded Subject’s statement |
| Diabetes Treatment | Questionnaire | PAst Medical REcords |
| Occupation | Questionnaire | Recorded Subject’s statement |
| Food | Questionnaire | Recorded Subject’s statement |
| CENTRAL OBESITY SCORE | MEASURED | Normal (male≤102cm/female≤88cm)  Obese(male>102cm/female88cm) |
| Abdominal Circumference | Measured | Waist circumference was measured 2.5 cm above the umbilicus |
| Fasting Plasma Glucose | Physical Examination | FPG was performed by finger prick method by using glucometer (Ascensia Entrust, Bayer Diagnostics, Tarrytown, NY, USA). |
| Systolic Blood Pressure | Physical Examination | Measurement of Systolic and Diastolic BP of registered subjects were recorded by a trained CHW with a mercury column sphygmomanometer (Deluxe, BPMR 120, Maharashtra, India) and a stethoscope in seated position after a resting period of 5 minutes. Mean of the two consecutive readings were recorded. |
| Diastolic Blood Pressure | Physical Examination |  |
| Duration of diabetes | Questionnaire | PAST MEDICAL RECORDS |
| Weight | Measured |  |
| Height | Measured |  |
| BMI | Calculated | Weight (kg)/height (m2) |
| Waist/Hip Ratio CO | Calculated | Waist/ Hip |
| ABDOMINAL CIRCUMFERENCE code | measured |  |
| Hip circumference | Measured | Hip circumference was measured at the greatest circumference around the buttocks. |
| Abdominal to Hip Ratio | Calculated | Abdominal/Hip |
| Duration of Diabetes treatment | Questionnaire | PAST MEDICAL RECORDS |

*- As defined in <https://knowindia.india.gov.in/profile/literacy.php>
