# Supplementary material for: Patterns of risk for diabetic retinopathy in the Mumbai slums: The Aditya Jyot Diabetic Retinopathy in Urban Mumbai Slums Study (AJ-DRUMSS) Report 3
Source: PLOS Glob Public Health. 2023 Apr 12;3(4):e0000351. doi: 10.1371/journal.pgph.0000351 (PMC10096465; doi:10.1371/journal.pgph.0000351)
Supplement: S6 Table — (DOCX) [file pgph.0000351.s006.docx]

Table S6. Variables collected for analysis of DR risk

| **Dichotomous Variables (18)** | Referent | Number of Individuals Missing Data |
| --- | --- | --- |
| Sex | Male | 0 |
| Hypertension | No | 3 |
| Smoking | No | 1 |
| Ear Lobe Crease | Absent | 109 |
| Polyuria | No | 0 |
| Polydypsia | No | 0 |
| Weight Loss | No | 0 |
| Ishemic Heart Disease | No | 1 |
| Stroke | No | 2 |
| Neuropathy | No | 1 |
| Nephropathy | No | 2 |
| Family History DM | No | 17 |
| Vegetarian Diet | Non-Vegetarian | 84 |
| Central Obesity Score | Normal (Female <0.85; Male <0.95) | 102 |
| Abdominal Circumference Code | Normal (Female; Male ≤ 102 cm) | 1 |
| Literate | Illiterate | 1 |
| Religion | Muslim | 0 – Christians removed from analysis |
| Treatment | No Metformin | 62 |
|  |  |  |
| **Categorical (2)** | **Categories** |  |
| Rice Wheat Eaters | Rice only (ref) | 102 |
|  | Both rice and wheat |  |
|  | Wheat only |  |
| Occupation | Working (ref) | 1 |
|  | Not Working |  |
|  | Housewife |  |
|  | Retired |  |
|  |  |  |
| **Continuous variables (13)** |  |  |
| Age |  | 0 |
| Fasting Plasma Glucose |  | 68 |
| Systolic BP |  | 1 |
| Diastolic BP |  | 1 |
| Duration of DM |  | 0 |
| Weight |  | 7 |
| Height |  | 9 |
| BMI |  | 15 |
| Waist Hip Ratio - Central Obesity |  | 102 |
| Abdominal Circumference |  | 1 |
| Hip circumference |  | 102 |
| Abdominal Hip Ratio* |  |  |
| Duration of DM treatment** |  |  |

* Removed from analysis due to high correlation with WHRCO

**Removed from analysis due to high correlation with Duration of Diabetes
