# Supplementary material for: Patterns of risk for diabetic retinopathy in the Mumbai slums: The Aditya Jyot Diabetic Retinopathy in Urban Mumbai Slums Study (AJ-DRUMSS) Report 3
Source: PLOS Glob Public Health. 2023 Apr 12;3(4):e0000351. doi: 10.1371/journal.pgph.0000351 (PMC10096465; doi:10.1371/journal.pgph.0000351)
Supplement: S7 Table — (DOCX) [file pgph.0000351.s007.docx]

| Supplemental Table 5. Univariate Analyses | | | | |  | |  |  |  |  |  |  |  |  |
| --- | --- | --- | --- | --- | --- | --- | --- | --- | --- | --- | --- | --- | --- | --- |
| Variable | **N** | **No DR** | **Yes Dr** |  | | Beta | Standard Error | Wald Chi-Square | P-value | Odds Ratio | 95% LCL | 95% UCL |  |  |
| **Type of Diabetic Retinopathy** |  |  |  |  | | 15.1069 | 3.5341 | 18.2724 | <.0001 | >999.999 | >999.999 | >999.999 | Complete separation  of data points detected. |  |
| No DR | 905 | 905 | 0 |  | |  |  |  |  |  |  |  | Warning: The  maximum likelihood  estimate does not exist. | The maximum likelihood estimate does not exist. |
| Mild NPDR | 106 | 0 | 106 |  | |  |  |  |  |  |  |  |  |  |
| Mild NPDR + DME | 6 | 0 | 6 |  | |  |  |  |  |  |  |  |  |  |
| Mod NPDR | 32 | 0 | 32 |  | |  |  |  |  |  |  |  |  |  |
| Mod NPDR + DME | 7 | 0 | 7 |  | |  |  |  |  |  |  |  |  |  |
| Severe NPDR | 48 | 0 | 48 |  | |  |  |  |  |  |  |  |  |  |
| Severe NPDR + DME | 38 | 0 | 38 |  | |  |  |  |  |  |  |  |  |  |
| PDR | 5 | 0 | 5 |  | |  |  |  |  |  |  |  |  |  |
| PDR + DME | 16 | 0 | 16 |  | |  |  |  |  |  |  |  |  |  |
| Missing | 0 | 0 | 0 |  | |  |  |  |  |  |  |  |  |  |
| **Macula Dx** |  |  |  |  | | 17.8096 | 6.6826 | 7.1025 | 0.0077 | >999.999 | 111.253 | >999.999 | Complete separation  of data points detected. |  |
| No DR | 905 | 905 | 0 |  | |  |  |  |  |  |  |  | Warning: The  maximum likelihood  estimate does not exist. | The maximum likelihood estimate does not exist. |
| Non Sight Threatening DR (NSTDR) | 138 | 0 | 138 |  | |  |  |  |  |  |  |  |  |  |
| Sight Threatening DR (STDR) | 120 | 0 | 120 |  | |  |  |  |  |  |  |  |  |  |
| Missing | 0 | 0 | 0 |  | |  |  |  |  |  |  |  |  |  |
| **Sex (Female)** |  |  |  |  | |  |  |  |  |  |  |  |  |  |
| Female | 618 | 509 | 109 |  | | -0.2818 | 0.0714 | 15.5888 | <.0001 | 0.569 | 0.43 | 0.753 |  |  |
| Male (ref) | 545 | 396 | 149 |  | |  |  |  |  |  |  |  |  |  |
| **Literacy** |  |  |  |  | |  |  |  | 0.0926 |  |  |  |  |  |
| Illiterate (ref) | 326 | 267 | 59 |  | |  |  |  |  |  |  |  |  |  |
| Literate | 813 | 619 | 194 |  | | 0.249 | 0.1973 | 1.5929 | 0.2069 | 1.418 | 1.025 | 1.962 |  |  |
| Can read not write | 23 | 19 | 4 |  | | -0.1487 | 0.3709 | 0.1607 | 0.6885 | 0.953 | 0.313 | 2.904 |  |  |
| Missing | 1 |  |  |  | |  |  |  |  |  |  |  |  |  |
| **Lit** |  |  |  |  | |  |  |  |  |  |  |  |  |  |
| Illiterate (ref) | 326 | 267 | 59 |  | |  |  |  |  |  |  |  |  |  |
| Literate | 836 | 638 | 198 |  | | 0.1698 | 0.0826 | 4.2214 | 0.0399 | 1.404 | 1.016 | 1.941 |  |  |
|  |  |  |  |  | |  |  |  |  |  |  |  |  |  |
| **Religion** |  |  |  |  | |  |  |  | 0.2945 |  |  |  |  |  |
| Hindu(ref) | 1009 | 785 | 224 |  | |  |  |  |  |  |  |  |  |  |
| Muslim | 137 | 104 | 33 |  | | 0.5769 | 0.3694 | 2.4398 | 0.1183 | 1.112 | 0.732 | 1.69 |  |  |
| Christian | 17 | 16 | 1 |  | | -1.0477 | 0.6909 | 2.3 | 0.1294 | 0.219 | 0.029 | 1.661 |  |  |
| Missing | 0 | 0 | 0 |  | |  |  |  |  |  |  |  |  |  |
| **Rel** |  |  |  |  | |  |  |  |  |  |  |  |  |  |
| Hindu (ref) | 1009 | 785 | 224 |  | |  |  |  |  |  |  |  |  |  |
| Muslim | 137 | 104 | 33 |  | | 0.0531 | 0.1068 | 0.2468 | 0.6193 | 1.112 | 0.732 | 1.69 |  |  |
|  |  |  |  |  | |  |  |  |  |  |  |  |  |  |
| **Occupation** |  |  |  |  | |  |  |  | 0.0199 |  |  |  |  |  |
| Working (ref) | 453 | 341 | 112 |  | |  |  |  |  |  |  |  |  |  |
| Not Working | 89 | 62 | 27 |  | | 0.3235 | 0.188 | 2.963 | 0.0852 | 1.326 | 0.805 | 2.187 |  |  |
| Retired | 89 | 68 | 21 |  | | -0.0206 | 0.1998 | 0.0107 | 0.9178 | 0.94 | 0.551 | 1.604 |  |  |
| Housewife | 531 | 434 | 97 |  | | -0.3439 | 0.1227 | 7.856 | 0.0051 | 0.681 | 0.501 | 0.925 |  |  |
| Missing | 1 |  |  |  | |  |  |  |  |  |  |  |  |  |
| **Smoking** |  |  |  |  | |  |  |  |  |  |  |  |  |  |
| No | 926 | 738 | 188 |  | | -0.2522 | 0.0821 | 9.4273 | 0.0021 | 0.604 | 0.438 | 0.833 |  |  |
| Yes (ref) | 236 | 166 | 70 |  | |  |  |  |  |  |  |  |  |  |
| Missing | 1 |  |  |  | |  |  |  |  |  |  |  |  |  |
| **Diagonal Ear Lobe Crease (ELC)** |  |  |  |  | |  |  |  |  |  |  |  |  |  |
| Absent | 966 | 763 | 203 |  | | -0.3324 | 0.1192 | 7.7796 | 0.0053 | 0.514 | 0.322 | 0.821 |  |  |
| Present (ref) | 88 | 58 | 30 |  | |  |  |  |  |  |  |  |  |  |
| Missing | 109 |  |  |  | |  |  |  |  |  |  |  |  |  |
| **Polyuria** |  |  |  |  | |  |  |  |  |  |  |  |  |  |
| No | 314 | 263 | 51 |  | | -0.2542 | 0.0863 | 8.6759 | 0.0032 | 0.601 | 0.429 | 0.8 |  |  |
| Yes(ref) | 849 | 642 | 207 |  | |  |  |  |  |  |  |  |  |  |
| Missing | 0 | 0 | 0 |  | |  |  |  |  |  |  |  |  |  |
| **Polydysia** |  |  |  |  | |  |  |  |  |  |  |  |  |  |
| No | 325 | 272 | 53 |  | | -0.254 | 0.0852 | 8.8998 | 0.0029 | 0.602 | 0.431 | 0.84 |  |  |
| Yes(ref) | 838 | 633 | 205 |  | |  |  |  |  |  |  |  |  |  |
| Missing |  |  |  |  | |  |  |  |  |  |  |  |  |  |
| **Weight Loss** |  |  |  |  | |  |  |  |  |  |  |  |  |  |
| No | 845 | 669 | 176 |  | | -0.1391 | 0.0768 | 3.2786 | 0.0702 | 0.757 | 0.56 | 1.023 |  |  |
| Yes (ref) | 318 | 236 | 82 |  | |  |  |  |  |  |  |  |  |  |
| Missing | 0 | 0 | 0 |  | |  |  |  |  |  |  |  |  |  |
| **IHD** |  |  |  |  | |  |  |  |  |  |  |  |  |  |
| No | 1057 | 821 | 236 |  | | 0.0406 | 0.1255 | 0.1045 | 0.7465 | 1.084 | 0.663 | 1.773 |  |  |
| Yes (ref) | 105 | 83 | 22 |  | |  |  |  |  |  |  |  |  |  |
| Missing | 1 |  |  |  | |  |  |  |  |  |  |  |  |  |
| **Stroke** |  |  |  |  | |  |  |  |  |  |  |  |  |  |
| No | 1132 | 885 | 247 |  | | -0.3919 | 0.1947 | 4.0504 | 0.0442 | 0.457 | 0.213 | 0.98 |  |  |
| Yes (ref) | 29 | 18 | 11 |  | |  |  |  |  |  |  |  |  |  |
| Missing | 2 |  |  |  | |  |  |  |  |  |  |  |  |  |
| **Neuropathy** |  |  |  |  | |  |  |  |  |  |  |  |  |  |
| No | 1125 | 880 | 245 |  | | -0.3328 | 0.1759 | 3.5779 | 0.0586 | 0.514 | 0.258 | 1.024 |  |  |
| Yes (ref) | 37 | 24 | 13 |  | |  |  |  |  |  |  |  |  |  |
| Missing | 1 |  |  |  | |  |  |  |  |  |  |  |  |  |
| **Nephropathy** |  |  |  |  | |  |  |  |  |  |  |  |  |  |
| No | 1138 | 889 | 249 |  | | -0.322 | 0.2218 | 2.1076 | 0.1466 | 0.525 | 0.22 | 1.253 |  |  |
| Yes (ref) | 23 | 15 | 8 |  | |  |  |  |  |  |  |  |  |  |
| Missing | 2 |  |  |  | |  |  |  |  |  |  |  |  |  |
| **Status of Hypertension** |  |  |  |  | |  |  |  |  |  |  |  |  |  |
| No | 551 | 436 | 115 |  | | -0.0757 | 0.0709 | 1.1383 | 0.286 | 0.86 | 0.651 | 1.135 |  |  |
| Yes (ref) | 609 | 466 | 143 |  | |  |  |  |  |  |  |  |  |  |
| Missing | 3 |  |  |  | |  |  |  |  |  |  |  |  |  |
| **Family History of DM** |  |  |  |  | |  |  |  |  |  |  |  |  |  |
| No | 700 | 546 | 154 |  | | -0.0187 | 0.0727 | 0.066 | 0.7973 | 0.963 | 0.725 | 1.281 |  |  |
| Yes (ref) | 446 | 345 | 101 |  | |  |  |  |  |  |  |  |  |  |
| Missing | 17 |  |  |  | |  |  |  |  |  |  |  |  |  |
| **COScore** |  |  |  |  | |  |  |  |  |  |  |  |  |  |
| Normal (Female<0.85/Male<0.95) | 200 | 150 | 50 |  | |  |  |  |  |  |  |  |  |  |
| Central Obesity (Female>=0.85/Male>=0.95 | 861 | 677 | 184 |  | | -0.1021 | 0.0916 | 1.241 | 0.2653 | 0.815 | 0.569 | 1.168 |  |  |
| Missing | 102 |  |  |  | |  |  |  |  |  |  |  |  |  |
| **Abdominal Circumference Code** |  |  |  |  | |  |  |  |  |  |  |  |  |  |
| Normal(male<=102cm/female<=88cm) (ref) | 572 | 409 | 163 |  | |  |  |  |  |  |  |  |  |  |
| Obese(male>102cm/female88cm) | 590 | 496 | 93 |  | | -0.3717 | 0.0729 | 26.02 | <.0001 | 0.476 | 0.357 | 0.633 |  |  |
| Missing | 1 |  |  |  | |  |  |  |  |  |  |  |  |  |
| **Vegeterian Eater** |  |  |  |  | |  |  |  |  |  |  |  |  |  |
| Vegeterian Eater | 108 | 86 | 22 |  | | -0.0619 | 0.1255 | 0.243 | 0.622 | 0.884 | 0.54 | 1.445 |  |  |
| Non-Vegetarian Eater | 971 | 753 | 218 |  | |  |  |  |  |  |  |  |  |  |
| Missing | 84 | 839 | 240 |  | |  |  |  |  |  |  |  |  |  |
| **Rice/Wheat Eater** |  |  |  |  | |  |  |  | 0.1492 |  |  |  |  |  |
| Rice | 27 | 22 | 5 |  | | -0.2112 | 0.3353 | 0.3968 | 0.5287 | 0.858 | 0.32 | 2.299 |  |  |
| Wheat | 227 | 166 | 61 |  | | 0.2693 | 0.1951 | 1.9052 | 0.1675 | 1.387 | 0.989 | 1.947 |  |  |
| Both Rice and Wheat | 807 | 638 | 169 |  | |  |  |  |  |  |  |  |  |  |
| Missing | 102 |  |  |  | |  |  |  |  |  |  |  |  |  |
| **Metformin** | |  |  |  | |  |  |  |  |  |  |  |  |  |
| No Metformin (Ref) | 332 | 260 | 72 |  | |  |  |  |  |  |  |  |  |  |
| Yes Metformin | 769 | 595 | 174 |  | | -0.0168 | 0.1022 | 0.027 | 0.8696 | 0.967 | 0.648 | 1.444 |  |  |
|  |  |  |  |  | |  |  |  |  |  |  |  |  |  |
| **Age** |  |  |  |  | | 0.00081 | 0.00759 | 0.0114 | 0.915 | 1.001 | 0.986 | 1.016 |  |  |
| **BP Systolic** | |  |  |  | | 0.00853 | 0.00308 | 7.6884 | 0.0056 | 1.009 | 1.003 | 1.015 |  |  |
| **BP Diastolic** | |  |  |  | | -0.00069 | 0.00581 | 0.0141 | 0.9054 | 0.999 | 0.988 | 1.011 |  |  |
| **DurDM** |  |  |  |  | | 0.1234 | 0.0126 | 96.3733 | <.0001 | 1.131 | 1.104 | 1.16 |  |  |
| **DurDMCode** | |  |  |  | | 0.7077 | 0.0744 | 90.3659 | <.0001 | 2.029 | 1.754 | 2.348 |  |  |
| **FPGValue** |  |  |  |  | | 0.00261 | 0.000833 | 9.8519 | 0.0017 | 1.003 | 1.001 | 1.004 |  |  |
| **DurRx** |  |  |  |  | | 0.0951 | 0.0131 | 53.0482 | <.0001 | 1.1 | 1.072 | 1.128 |  |  |
| **Weight** |  |  |  |  | | -0.0114 | 0.00591 | 3.6834 | 0.055 | 0.989 | 0.977 | 1 |  |  |
| **Height** |  |  |  |  | | 0.0132 | 0.00666 | 3.9119 | 0.0479 | 1.013 | 1 | 1.027 |  |  |
| **BMI** |  |  |  |  | | -0.0446 | 0.0153 | 8.4481 | 0.0037 | 0.956 | 0.928 | 0.986 |  |  |
| **WHRCO** |  |  |  |  | | -0.8392 | 0.6875 | 1.4898 | 0.2223 | 0.432 | 0.112 | 1.663 |  |  |
| **Abdcircum** |  |  |  |  | | -0.017 | 0.00575 | 8.6936 | 0.0032 | 0.983 | 0.972 | 0.994 |  |  |
| **Hipcircum** |  |  |  |  | | -0.00955 | 0.00691 | 1.9095 | 0.167 | 0.99 | 0.977 | 1.004 |  |  |
| **AHRatioBase** |  |  |  |  | | -0.9759 | 0.6675 | 2.1374 | 0.1437 | 0.377 | 0.102 | 1.394 |  |  |
